# The sense of agency shapes body schema and peripersonal space

**DOI:** 10.1038/s41598-018-32238-z

**Published:** 2018-09-14

**Authors:** Mariano D’Angelo, Giuseppe di Pellegrino, Stefano Seriani, Paolo Gallina, Francesca Frassinetti

**Affiliations:** 1 Department of Psychology, University of Bologna, Bologna, Italy; 2 CsrNC, Centre for Studies and Research in Cognitive Neuroscience, University of Bologna, Bologna, Italy; 3 Istituti Clinici Scientifici Maugeri, IRCCS, via Maugeri 4, Pavia, Italy; 4 School of Psychology, Bangor University, Bangor, UK; 5 Department of Engineering and Architecture, University of Trieste, Trieste, Italy

## Abstract

Body schema, a sensorimotor representation of the body used for planning and executing movements, is plastic because it extends by using a tool to reach far objects. Modifications of peripersonal space, i.e., a functional representation of reach space, usually co-occur with body schema changes. Here, we hypothesized that such plastic changes depend on the experience of controlling the course of events in space trough one’s own actions, i.e., the sense of agency. In two experiments, body schema and peripersonal space were assessed before and after the participants’ sense of agency over a virtual hand was manipulated. Body schema and peripersonal space enlarged or contracted depending on whether the virtual hand was presented in far space, or closer to the participants’ body than the real hand. These findings suggest that body schema and peripersonal space are affected by the dynamic mapping between intentional body movements and expected consequences in space.

## Introduction

The term body schema refers to a sensorimotor representation of the body morphology used for planning and executing body movements. This sensorimotor body representation entails tracking and updating the position and configuration of the body parts in space at the service of action^1–3^. A main characteristic of the body schema is that it is highly plastic. For example, there is abundant evidence that the active use of a tool, to interact with objects placed beyond one’s reaching space, changes the body schema, increasing length of the sensorimotor representation of the arm^4–6^. Body schema has been found to be sensitive also to other manipulations. For instance, after right arm immobilization, participants overused the remaining free arm, modulating its representation. As a consequence, participants perceived their overused limb as longer after, than before, immobilization^7^. In another study, Tajadura-Jimenez *et al*.^8^ found that body schema is affected by the sound of one’s action. Body schema extended after participants tapped on a surface and listened to a tapping sound originating at a double the distance at which they actually tapped. This evidence emphasizes the strictly connection between body schema, space representation and the motor system.

To this regard, body schema is closely interwoven with a functional representation of the action space immediately surrounding the body, the so-called peripersonal space^9^. Peripersonal space representation depends on the activity of multisensory neurons in fronto-parietal network, including the premotor cortex (PMc) and the posterior parietal cortex (PPc). These neurons respond both to tactile stimuli on body parts and to visual and/or auditory stimuli presented near the same body part. Peripersonal space has not only a sensory function, but also motor function. Indeed, in monkeys fronto-parietal multisensory neurons control movements of the head, arm and hand towards or away from nearby objects^10,11^. In humans, auditory or visual stimuli presented in the peripersonal space modulate the excitability of the hand representation in the motor cortex^12,13^. Indeed, when we perform an action, the motor system needs to compute target positions relative to head or hand. Thus, peripersonal space is a multisensory-motor interface that may serve to encode the position of sensory stimuli to generate goal directed action toward objects within the reaching distance^9,14^. As body schema, also peripersonal space is highly modulated by several action-dependent manipulations. Far space becomes included into the peripersonal space after tool-use manipulation^15,16^. Moreover, near space representation is contracted when participants perform action while wearing weights applied to their wrist^17^. Thus, the possibility to act in space is critical for the construction of both body and space representations.

Setting out from these premises, in the present study, we hypothesized that body schema and peripersonal space extent depend on the experience of controlling the course of events in space trough one’s own actions.

In cognitive neuroscience, the sense of controlling one’s own motor acts and, through them, the events in the external environment, has been termed sense of agency^18,19^. By definition, the sense of agency depends on the mental association between an intentional action and its sensory outcome. Thus, while sense of agency begins with the sensorimotor experience of controlling one’s own body, humans can learn new contingent associations between movements and outcome, transferring a sense of agency from one’s own limb to objects or events, external to the body. Several studies have shown that it is possible to retain a sense of agency over an external object without necessarily perceive it as belonging to our body (i.e. the body ownership)^20–25^. However, although the relationship between body ownership and the sense of agency has been extensively addressed, there are relative few studies that investigate whether and how body schema and peripersonal space may change when we actively control external events through one’s own action. Specifically, here we tested the hypothesis that body schema and peripersonal space representations are the consequence of the simple associations between one’s own intentional actions and their outcomes occurring in space (i.e., the sense of agency).

Body schema was assessed through the *Forearm bisection task*. In this task, participants are asked to point at their forearm midpoint, a paradigm widely used to assess changes in body metric representation^26–28^. For instance, it has been recently shown that the perceived arm midpoint shifted distally after the active use of the tool, consistent with an increase of the perceived length of the arm representation^6^. In the current work, the *Forearm bisection task* was performed before and after the participant’s sense of agency over a far virtual hand was manipulated. To this aim, an infrared motion captured device was used to track in real time the participant’s hand movements, and control a virtual hand presented on a PC screen placed beyond reachable space. If body schema is sensitive to the experience of controlling far space through one’s own actions, we should expect that participants pointed to their forearm midpoint more distally after sensing agency for the far virtual hand. Moreover, given the close functional relationship with body schema^29^, we also tested how peripersonal space changes as a function of agency manipulation. To do so, we measured peripersonal space through a *Reaching distance estimation task*^30–32^, in which participants were asked to stop a ball, either approaching or withdrawing from them, at a distance at which they thought they could reach it by extending the arm. Since body schema and peripersonal space are functionally linked, we expected that peripersonal space, too, extended following agency manipulation.

## Methods

### Participants

Twenty-four participants (12 females), volunteered for the study (age range = 20–28; mean age = 23.86).

Sample size was determinate a priori by conducting a power analysis using G*Power 3^33^ specifying a medium effect size (*η*^2^_*p*_ = 0.25). Within our chosen sample size and effect size, the power (1-β) was approximately 0.80. Participants were naive to the experimental hypothesis and had no self-reported history of neurological or psychiatric disease. All, but one, were right-handed, as assed by Edinburgh Handedness Inventory^34^. They provided written informed consent to participate in the experiment. All methods, approved by the Ethical Committee of the University of Bologna, were conducted in accordance with the 2008 Helsinki Declaration.

### Procedure

Participants were comfortably seated during the experiment in front of a table. They performed a *Forearm bisection task*, in order to assess the sensorimotor representation of the arm length. Participants sat blindfolded with their left and right forearms (from the elbow to fingertips) on the table, positioned at about 30 cm from the midsagittal plane. On each trial (15 in total), they were instructed to indicate, with the left index finger, the midpoint of their right forearm, considering the elbow and the tip of the middle finger as the two extremities. A transparent screen (10.5 cm in height) was placed over the right forearm to avoid tactile feedback. The subjective midpoint was measured with a digital laser meter (Agatec DM100, error ± 3 mm) as the distance between the elbow, corresponding to the 0 mm, and the indicated point.

**Figure 1:**
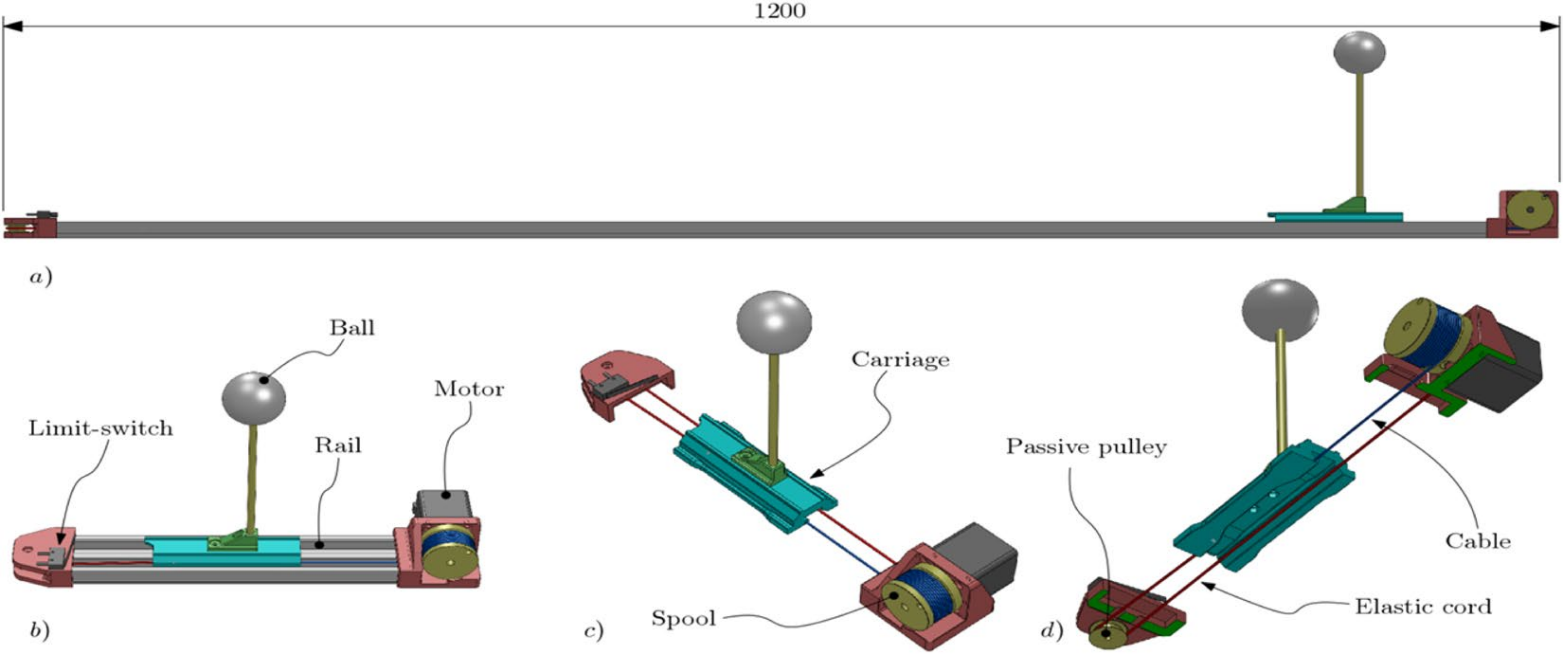
The present linear actuator is based on a motorized spool that manoeuvres the carriage by means of a cable and by an elastic element opposite to the cable. When the motor rotates clockwise, the cable is pulled by the spool and this causes the carriage to move in the direction of the motor and the elastic cord is placed into tension. When the rotation of the motor is reversed, the cable is slowly released from the spool, and the tensioned elastic cord provides the force for the carriage to move away from the motor. This type of linear actuator was used mainly for safety reasons: it is a passively safe device, since the only force produced directly towards the participant is given by the elastic cord, and not by the motor itself. In (**a**) a side view of the apparatus is shown; in (**b**) a reduced length version is shown for clarity; in (**c**) the same short version is shown, this time without the rails, in order to show the complete carriage and part of the cable and elastic cord which drive it; in (**d**) the same version is shown from under the top plane: a cross-section (shown in green) was executed in the illustration to show the passive pulley and the complete path of the cable and elastic cord.

In addition, peripersonal space was assessed by a *Reaching distance estimation task*. In this task, a small ball (40 mm in diameter), controlled by the experimenter through a linear actuator, moved at constant speed (26,93 mm/s) towards or away from the participants (approaching vs. withdrawing trials). The ball starting position was at 33 cm from the participants’ sternum in the withdrawing trials, and at 133 cm in the approaching trials. Participants kept the hands over the table, resting only the wrists over it. They were asked to estimate reaching distance (“stop the ball at the distance you think you can reach it by an extension of your arm”). Participants could fine-tune the ball distance by asking the experimenter to move the ball slightly further or forward. Finally, between trials, they were asked to close their eyes. The task was repeated for 15 trials, counterbalancing approaching and withdrawing trials. To control the ball movement, we used the aforementioned linear actuator (see Fig. 1). The present linear actuator is operated by a ATmega328P microcontroller with a dedicated firmware; an LCD display was used to report the distance, in millimeters, from the zero-position of the carriage. The drive speed can be adjusted via a potentiometer from 0 to 27 mm/s approximately; two buttons provide forward and backwards motion and a calibration procedure (zeroing) for the machine is performed by a third button. The linear actuator was shielded from view with an opaque barrier to prevent participants from using it as a frame of reference.

**Figure 2:**
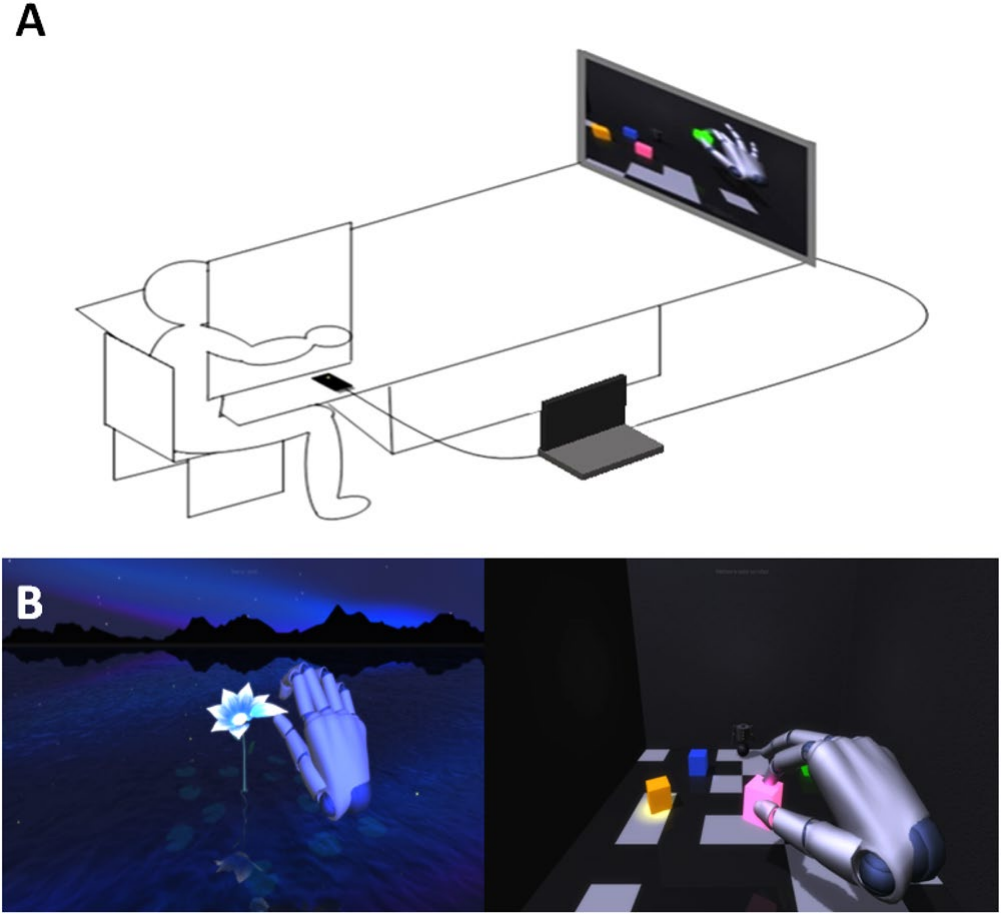
(**A**) Leap motion training: we placed a leap motion controller near the palm of the right hand and a screen at approximately 140 cm from the participants’ sternum. Both the leap motion controller and screen were connected to a PC, so that leap motion controller was used to track in real time the participant’s hand movements in order to control a 3D virtual hand projected on the screen. (**B**) Screenshots of two tasks performed by participants during leap motion training (V2 Playground app).

The order of two tasks was counterbalanced across participants, who performed them twice, before and after a 15 minutes training aimed at experiencing a sense of agency for a 3D virtual hand. An infrared motion capture device, i.e. leap motion controller, which tracked in real time the participant’s hand movement, was used to control the 3D virtual hand presented on a PC screen placed at approximately 140 cm from the participants’ sternum. Leap motion controller^35^ is an infrared motion capture device designed for hand tracking in virtual reality, consisting of two cameras and three infrared LEDs. Thanks to its wide-angle lenses (the field of view is 150° wide and 120° deep), the device has a large interaction 3D space of eight cubic feet. Both the leap motion controller and the screen were connected to a PC, such that when participants moved their right hand, a virtual hand, projected on the screen, moved synchronously with the participant’s real right hand (See Fig. 2A). At the beginning of the leap motion training, participants were asked to watch the screen and to raise their right arm in order to perform a game. Participants were instructed to virtually grasp objects and make precision grip by controlling the virtual hand (See Fig. 2B). We positioned a barrier on the table, along the participant’s right shoulder, to hide both the participant’s real hand and the leap motion controller.

The training consisted of two timing conditions: synchronous and asynchronous visual feedback. In the synchronous condition, participants were shown virtual hand movements responding in real time to their own right hand movements. In the asynchronous condition, 3-second delay was interposed between the participant’s real hand and the virtual hand movements. The order of the synchronous and asynchronous conditions was counterbalanced across participants, who performed both in two different days.

At the end of the experimental session, participants were asked to complete a 12-statements questionnaire to assess the ownership and agency sensed over the 3D virtual hand, using a 7-point Likert scale ranging from - 3 (strongly disagree) to 3 (strongly agree). Three statements referred to the feeling of ownership, and three statements described sensations related to agency. The remaining six statements served as control for suggestibility, task compliance and expectancy effect, and were adapted from previous studies^20,21^. Three statements served as control for ownership and three for agency (See Table 1).

**Table 1:** Statements used in the questionnaire.

Questionnaire’s statements	
Ownership	I felt as if I was looking at my own hand
	I felt as if the virtual hand was part of my body
	I felt as if the virtual hand was my hand
Ownership control	It seems as if I had more than one right hand
	It felt as if I had no longer a right hand, as if my right hand had disappeared
	I felt as my real hand was turning virtually
Agency	I felt as if I could cause movements of the virtual hand
	I felt as if I could control movements of the virtual hand
	The virtual hand was obeying my will and I can make it move just like I want it
Agency control	I felt as if the virtual hand was controlling my will
	It seemed as if the virtual hand had a will of its own
	I felt as if the virtual hand was controlling me

### Results

For each task, a repeated measure ANOVA was performed on the mean distances, with Timing (Synchronous and Asynchronous conditions) and Session (Pre and Post leap motion training) as within-subjects factors. Significant interactions were analysed by Newman-Keuls post-hoc test.

**Figure 3:**
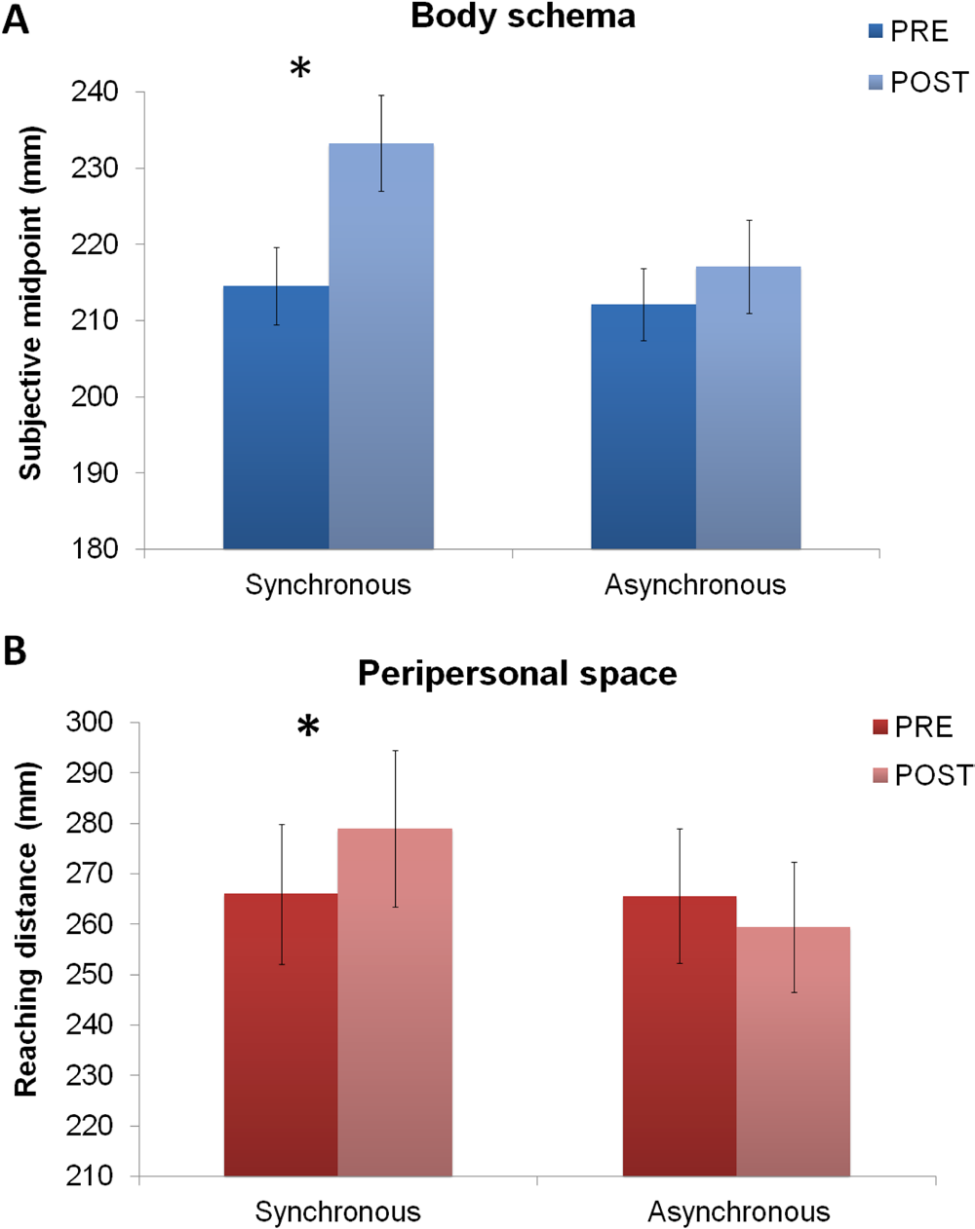
Effects of leap motion training with the virtual hand presented farther forward than actual hand location. (**A**) Effects of leap motion training on forearm bisections. The graph shows the statistical comparison of mean distances from the elbow to the indicated forearm midpoint (in mm) as a function of Timing condition (Synchronous and Asynchronous leap motion training) and Session (Pre and Post leap motion training). Asterisk indicates a significant difference in forearm bisections between pre and post leap motion synchronous training. Error bars indicate standard error of the mean. (**B**) Effects of leap motion training on the Reaching distance estimation task. The graph shows the average reaching distance (in mm) as a function of the Timing condition (Synchronous and Asynchronous leap motion training) and Session (Pre and Post leap motion training). Asterisk indicates a significant difference in the reaching judgments between pre and post synchronous leap motion training. Error bars indicate standard error of the mean.

In the *Forearm bisection task*, the ANOVA showed a main effect of Session (F_1,23_ = 12.38; p < 0.005; *η*^2^_*p*_ = 0.34) and, crucially, a significant interaction between Timing and Session (F_1,23_ = 10.72; p < 0.005; *η*^2^_*p*_ = *0.31*). Post hoc tests indicated that the interaction was driven by the fact that, following leap motion synchronous training, participants indicated the subjective forearm midpoint more distally as compared to the pre-training (214 vs. 233 mm; p < 0.001). In contrast, there was no significant difference in the subjective midpoint estimates between pre and post leap motion asynchronous training (212 vs. 217 mm; p = 0.23; See Fig. 3A).

In the *Reaching distance estimation task*, the ANOVA revealed an interaction between Timing and Session (F_1,23_ = 9.07; p < 0.05; *η*^2^_*p*_ = 0.28). Post hoc test showed that the reaching distance was significantly extended after, as compared to before, the leap motion synchronous training (266 vs. 279 mm; p < 0.01). In contrast, the mean reaching distance was not significantly different between pre and post leap motion asynchronous training (265 vs. 259 mm; p = 0.23; See Fig. 3B).

Since peripersonal space and body schema are not completely interdependent constructs, as they can dissociate in some circumstances^7^, we tested whether the observed effect in one task predicted the observed effect in the other task. Thus, we conducted an ANCOVA on the forearm midpoint estimates prior and following synchronous training, with Session (Pre and Post) as a within factor. To control for the influence of peripersonal space, we entered the difference between post and pre reaching distance in the synchronous condition as a covariate in the analysis. Crucially, the difference in forearm midpoint estimates between pre and post synchronous training remained significant, even when controlling for the effect on peripersonal space (F_1,22_ = 22,92; p < 0.00005; *η*^2^_*p*_ = 0.51), thereby suggesting some degree of functional separation between body schema and peripersonal space representation.

In order to assess the sense of agency and ownership for the virtual hand, we computed a mean score from each of the three ownership statements, and a mean score from the three agency statements. Similarly, we computed average scores of the corresponding control statements. In this way, four single scores were computed: “Ownership”; “Agency”; “Ownership control” and “Agency control”. We interpreted a category as rated positively, or affirmed, when the average score was equal or higher than +1, indicating that at group level, the participants affirmed the experience of ownership or agency (this criterion has been used before^20,21,36^). Because Shapiro-Wilk test showed that the questionnaire data were not normally distributed (p > 0.05), we used the non-parametric Wilcoxon test for pairwise comparisons.

**Figure 4:**
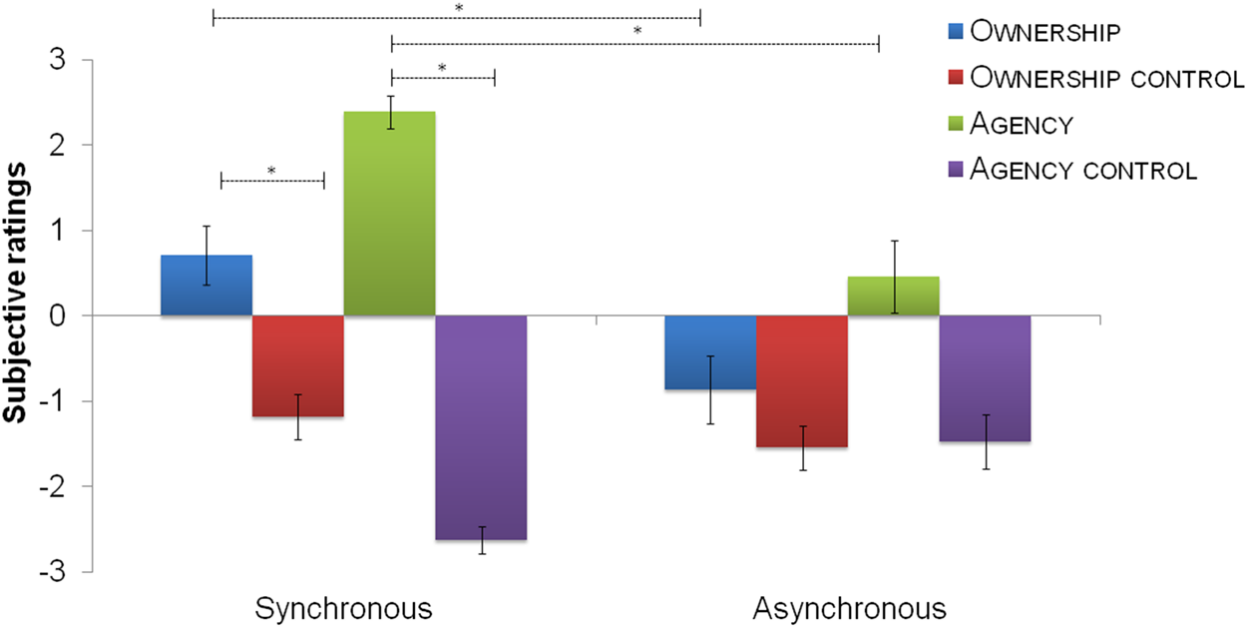
Subjective ratings of agency and ownership over the virtual far hand. The graph shows the average ownership, ownership control, agency, agency control ratings on a 7-point Likert scale as a function of the Timing condition (Synchronous and Asynchronous). Sense of agency was present in the Synchronous condition (>1). In contrast, sense of ownership was not positively affirmed. Asterisk indicates that agency category score (the mean of the three statements related to agency) was significantly greater than its respective control category in the synchronous condition, and greater in the synchronous than asynchronous condition. Error bars indicate standard error of the mean.

The average score was higher than +1 only for “Agency” in the synchronous condition (Average: 2.4). The “Agency” category in the synchronous condition was significantly different from its control category (“Agency control”, Z = −4.345; p < 0.0001), and from “Agency” in the asynchronous condition (Z = −3.526; p < 0.001), suggesting that participants experienced agency only during the training in which their own hand’s and virtual hand’s movements were synchronized (See Fig. 4). Moreover, in the synchronous condition, there was also a significant difference between agency and ownership ratings (Z = −3.86; p < 0.001), indicating that participants affirmed more strongly agency statements than ownership statements. Although “Ownership” category was not positively affirmed (Average: 0.71) in the synchronous condition, it was significantly different both from its control category (Z = −3.21; p < 0.005) and from “Ownership” in the asynchronous condition (Z = −3.77; p < 0.005).

Finally, we examined the influence of agency and ownership ratings on the effects found on body schema and peripersonal space after synchronous training. Thus, two ANCOVAs for each task were performed with Session (pre and post) as within-subjects factor. To control for the influence of the agency and ownership sensed for the virtual hand, we entered first “Agency” and then “Ownership” category scores as covariates in the analyses. In the *Forearm bisection task* and *Reaching distance task*, the difference between pre and post synchronous training in the forearm midpoint (F_1,22_ = 1,63; p = 0.21; *η*^2^_*p*_ = 0.070), and reaching distance estimates (F_1,22_ = 6.04; p < 0.05; *η*^2^_*p*_ = 0.21), respectively, were no longer significant after controlling for the “Agency” ratings. By contrast, the difference between pre and post forearm midpoint estimates (F_1,22_ = 10.86 p < 0.005; *η*^2^_*p*_ = 0.331), and between pre and post reaching distances (F_1,22_ = 0.51; p = 0.48; *η*^2^_*p*_ = 0.23), remained significant even after controlling for the influence of the “Ownership” scores, thereby suggesting that sense of agency, rather than ownership, plays a critical role in mediating the effect of synchronous training on body schema and peripersonal space representations.

In sum, the main purpose of the Experiment 1 was to investigate whether the sensorimotor representation of the body can be modulated by manipulating agency sensed over an external object. The results showed that participants experienced agency, explicitly assessed through a questionnaire, over a virtual hand that moved synchronously with their own hand movements. Crucially, experiencing agency for the virtual hand, projected in far space, significantly extended the representation of the arm. On the contrary, when virtual hand and participants’ hand movements were asynchronous, and participants did not report any sense of agency at the questionnaire, no significant modulation of the body schema was found. Moreover, as hypothesized, peripersonal space followed the same trend of results. Participants showed a significant enlargement of peripersonal space, as assed by the reaching distance estimation task, only in the synchronous condition, when they reported agency over the virtual hand. In contrast, after asynchronous condition, participants did not affirm agency for the virtual hand and any significant modulation of peripersonal space was found. Rather, our results suggest that body schema and peripersonal space are sensitive to the experience of controlling the course of events in space through one’s own actions. When participants controlled the virtual hand by moving their own hand, limb movements and their outcomes occurred synchronously but in different spatial positions. We speculate that the spatial mismatch between intentional movements and outcomes leads to the updating of the dimension of both body schema and peripersonal space. To further test this hypothesis we conducted Experiment 2 aimed to reveal the opposite modulation of Experiment 1, presenting the virtual hand behind the participant’s real hand, and closer to the subjects’ body than the real hand. Accordingly, when outcomes of the action are closer to the body than the action itself, we should find a contraction of both body schema and peripersonal space.

### Experiment 2

#### Participants

A new sample of twenty-four participants (12 females), volunteered for the study (age range = 20–28; mean age = 22.37). Participants were naive to the experimental hypothesis and had no self-reported history of neurological or psychiatric disease. All but two were right-handed, as assed by Edinburgh Handedness Inventory. They provided written informed consent to participate in the experiment. All methods, approved by the Ethical Committee of the University of Bologna, were conducted in accordance with the 2008 Helsinki Declaration.

#### Procedure

In Experiment 2, participants performed the *Forearm bisection task*, to assess body schema modulation, and the *Reaching distance estimation task*, to assess peripersonal space, before and after 15 minutes of synchronous and asynchronous leap motion training to modulate sense of agency for the 3D virtual hand.

The experimental procedure was identical of Experiment 1, with the exception of the distance at which the virtual hand was presented during the training. The PC screen, in which the virtual hand was projected, was placed at approximately 14 cm from the participant’s sternum. Also in Experiment 2, participants performed both synchronous and asynchronous training. The order of two timing conditions was counterbalanced across participants, who performed both in two different days. At the end of the experimental session, participants were asked to complete a 12-statements questionnaire to assess the ownership and agency experienced in relation to the 3D virtual hand.

## Results

**Figure 5:**
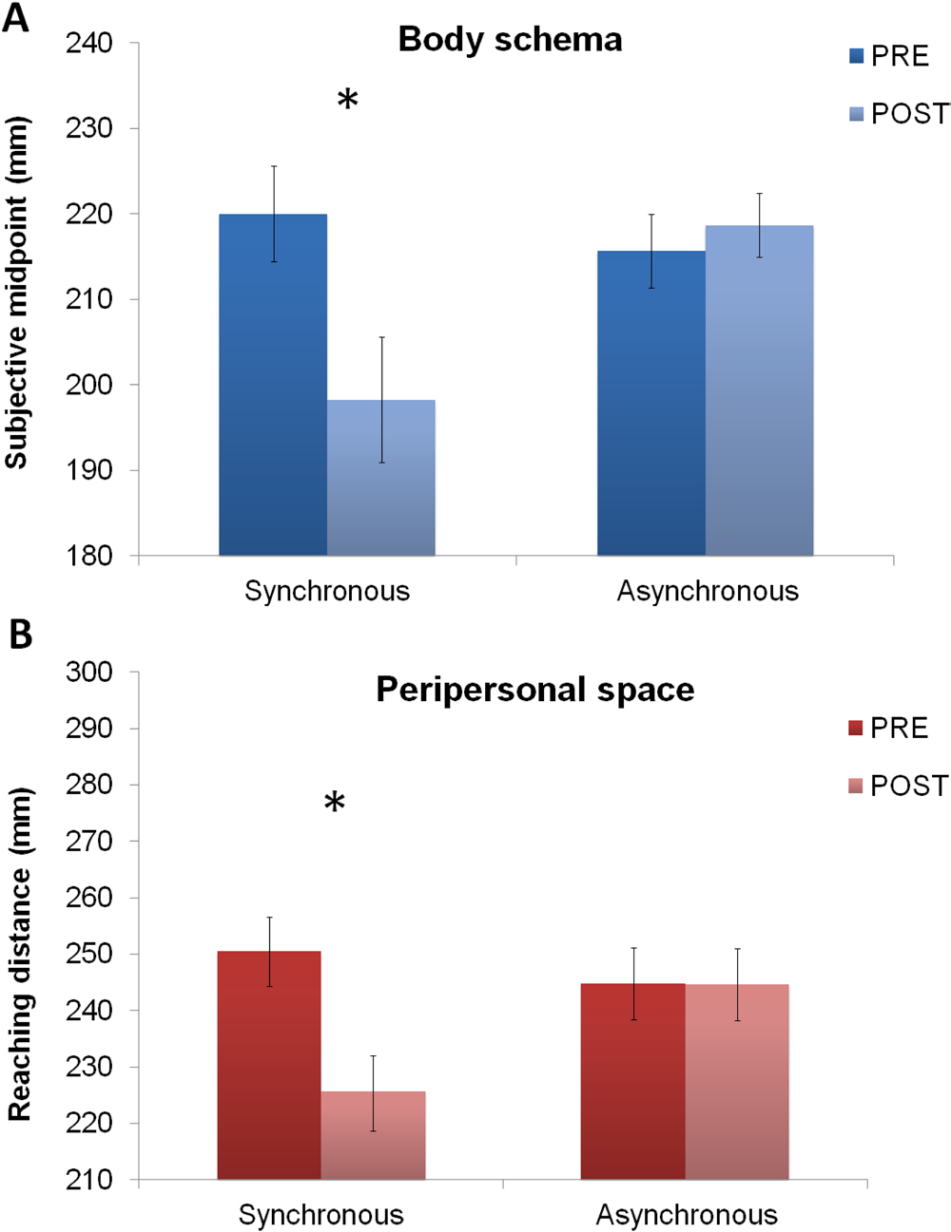
Effects of leap motion training with the virtual hand presented closer to the participants than actual hand location. (**A**) Effects of leap motion training on Forearm bisections. The graph shows the statistical comparison of mean distances from the elbow to the indicated forearm midpoint (in mm) as a function of the Timing condition (Synchronous and Asynchronous leap motion training) and Session (Pre and Post Leap motion training). Asterisk indicates a significant difference in forearm bisections between pre and post leap motion synchronous training. Error bars indicate standard error of the mean. (**B**) Effects of leap motion training on the Reaching distance estimation task. The graph shows the average reaching distance (in mm) as a function of the of the Timing condition (Synchronous and Asynchronous leap motion training) and Session (Pre and Post Leap motion training). Asterisk indicates a significant difference in the reaching judgments between pre and post synchronous leap motion training. Error bars indicate standard error of the mean.

In the *Forearm bisection task*, an ANOVA with Timing (Synchronous and Asynchronous conditions) and Session (Pre and Post leap motion training) as within-subjects factors, showed a main effect of Session (F_1,23_ = 11.45; p < 0.003; *η*^2^_p_ = 0.33) and, crucially, a significant interaction between Timing and Session (F_1,23_ = 18.65; p < 0.0003; *η*^2^_p_ = 0.44). Post hoc tests indicated that the interaction was driven by the fact that, following leap motion synchronous training, participants indicated the subjective forearm midpoint more proximally as compared to the pre-training (220 vs. 198 mm; p < 0.0003). In contrast, there was no significant difference in the subjective midpoint estimates between pre and post leap motion asynchronous training (216 vs. 219 mm; p = 0.44; See Fig. 5A).

In the *Reaching distance estimation task*, the ANOVA revealed an interaction between Timing and Session (F_1,23_ = 23.30; p < 0.0001; *η*^2^_*p*_ = 0.50). Post hoc test showed that the reaching distance was significantly reduced after as compared to before leap motion synchronous training (226 vs. 250 mm; p < 0.0001). In contrast, the mean reaching distance was not significantly different between post and pre leap motion asynchronous training (245 vs. 245 mm; p = 0.95; See Fig. 5B).

Also for the Experiment 2, we tested whether the observed effect on reaching space predicted the observed effect on the body schema. We performed an ANCOVA on the forearm midpoint with Session (Pre vs. Post) as within-subjects factor and the difference between post and pre reaching distance in the synchronous condition, as a covariate. Crucially, also in Experiment 2, there was still a significant difference in the subjective midpoint estimates between pre and post synchronous training (F_1,23_ = 0.310; p < 0.05; *η*^2^_p_ = 0.310), even after taking into account the effect on the reaching space as a covariate.

**Figure 6:**
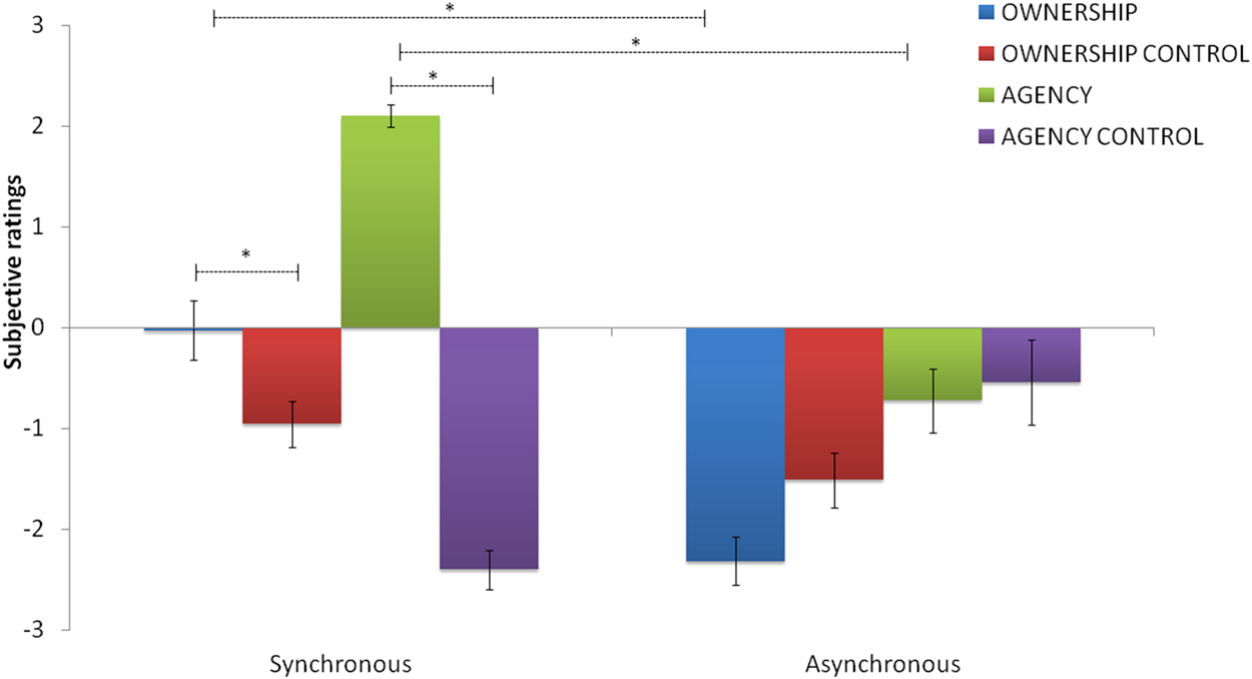
Subjective ratings of agency and ownership over the virtual near hand. The graph shows the average ownership, ownership control, agency, agency control ratings on a 7-point Likert scale as a function of the Timing condition (Synchronous and Asynchronous). Sense of agency was present in the Synchronous condition (>1). In contrast, sense of ownership was not positively affirmed. Asterisk indicates that agency category score (the mean of the three statements related to agency) was significantly greater than its respective control category in the synchronous condition and grater in the synchronous than asynchronous condition. Error bars indicate standard error of the mean.

Questionnaire data, analysed as in Experiment 1, showed that the average score was higher than + 1 only for “Agency” in the synchronous condition (Average: 2.09). The “Agency” category in the synchronous condition was significantly different from its control category (“Agency control”, Z = −4.20; p < 0.00001) and from “asynchronous Agency” (Z = −4.11; p < 0.00001), suggesting that participants experienced agency only during the training in which their own hand’s and virtual hand’s movements were synchronous (See Fig. 6). Moreover, in the synchronous condition, there was also a significant difference between “Agency” and “Ownership” ratings (Z = −4.28; p < 0.0001), indicating that participants affirmed more strongly agency statements than ownership statements. Also this time, “Ownership” category was not positively affirmed (Average: −0.02) in the synchronous condition, but it was significantly different both from its control category (Z = −2.02; p = 0.04) and from “Ownership” in the asynchronous condition (Z = −3.28; p < 0.05).

Thus, also in the Experiment 2, we examined the influence of “Agency” and “Ownership” ratings on body schema and peripersonal space changes after synchronous training with the virtual hand. Thus, we performed two ANCOVAs for each task, with Session (pre and post) as within-subjects factor. We entered first “Agency” and then “Ownership” category scores as covariates in the analysis.

As in Experiment 1, in the *Forearm bisection task* and *Reaching distance estimation task*, the difference between pre and post synchronous training in the forearm midpoint (F_1,22_ = 0.529; p = 0.47; *η*^2^_*p*_ = 0.023), and in the reaching distances (F_1,22_ = 2.84; p = 0.10; *η*^2^_*p*_ = 0.11), respectively, failed to reach significance after controlling for the “Agency” scores. By contrast, the difference between pre and post forearm midpoint estimates (F_1,22_ = 17.52 p < 0.0005; *η*^2^_*p*_ = 0.44), and between pre and post reaching distances (F_1,22_ = 24,95; p < 0.005; *η*^2^_p_ = 0.51), remained significant even after controlling for the influence of the “Ownership” scores.

Experiment 2 shows that it is possible to induce a contraction of both body schema and peripersonal space, presenting the virtual hand, over which participants experienced agency, behind their real hand. After experiencing agency for the virtual hand, participants indicated their forearm midpoint more proximally, as if their arm was shorter. In the same way, participants showed a significant reduction of peripersonal space, as assed by the *Reaching distance estimation task*, only in the synchronous condition, when they reported agency over the virtual hand.

## Discussion

In the present study, we induced changes in the extent of body schema and peripersonal space by manipulating the sense of agency over an external object. As recently suggested, to retain a vivid sense of agency, three conditions need to occur: first an internal volition that provides an experience of intentional action, second the occurrence of a body movement and third the external outcome of the action^37^. Humans, and other primates, can learn and exploit new intention-movements-outcome associations, transferring a sense of agency from one’s own limb to objects or events, external to the body^20–25^.

Experiment 1 showed that the sense of agency for a virtual hand projected in the far space extends both the body schema and peripersonal space, reproducing plastic modulations similar to those classically found after tool use. These results suggest that body schema and peripersonal space are concurrently modulated when agents establish new intention-movements-outcome associations to control events in the external environment through one’s own actions. Experiment 2 further supported this hypothesis by revealing, a concurrent contraction of both body schema and peripersonal space in healthy participants, when the virtual hand was presented closer to the body than their real hand. Moreover, participants, at the group level, did not rate positively ownership statements for the virtual hand. On the contrary, agency statements were strongly affirmed. This is in line with previous work^20,21,38^ showing that the sense of agency is partially dissociable from body ownership, and it is possible to retain a sense of agency over an external object without necessarily perceive it as belonging to our body. In Kalckert and Ehrsson^20,21^, for instance, the dissociation between body ownership and agency was investigated by using a modified version of the rubber hand illusion. In the classical rubber hand illusion^39^, touching a fake hand in synchrony with participant’s hand induces participants to perceive the rubber hand as part of their body. In Kalckert and Ehrsson version^20,21^, the participant’s index finger was connected to the rubber hand’s finger by a wooden stick. Thus, when participant moved his or her index finger, the rubber hand’s finger moved synchronously with respect to the participant’s movement. Crucially, when the rubber hand was placed in far location, or in an anatomical incongruent position with respect to the participant’s real hand, participants still reported a clear feeling of agency for the fake hand, even if they did not have anymore the illusion that the fake hand belonged to their body, as in the classical rubber hand illusion. Indeed, different studies with the rubber hand illusion have showed that the strength of the ownership illusion is constrained by the anatomical characteristic and spatial reference frames of the limb^40–42^. Note, however, that since in the current study we do not have implicit physiological measure of body ownership, we cannot completely rule out the possibility that participants did not experience ownership for the virtual hand. In fact, a significant difference in ownership ratings was found between synchronous and asynchronous conditions. Nevertheless, our findings show that, when agency ratings were added as covariates, pre and post synchronous training differences in forearm midpoint and reaching distance estimates were no longer apparent, providing support in favour of our hypothesis that the sense of agency, rather than the sense of ownership, plays a major role in the construction of body schema and peripersonal space representations.

This conclusion is in line with a relatively recent study demonstrating that the (illusory) ownership of a long or short arm *per se* is not enough to rescale distances in space^43^. Linkenauger *et al*.^43^, by using virtual reality technology, induced in participants the illusion of having a long or short virtual arm. They found that the distances to targets appeared closer when their virtual arm was long, compared to when their virtual arm was short, but only following a reaching experience. Crucially, modulations in space perception only occurred after participants actively performed reaching movements, thereby receiving sensory feedback from those movements.

This finding nicely fits with the results of present study. In both experiments, indeed, body schema and peripersonal space were updated when the consequences of the action occurred synchronously with participant’s movements, but in a different spatial position than expected, based on the actual hand position. This spatial mismatch caused a modulation in body schema and peripersonal space, suggesting that these representations emerge from the precise and dynamic mapping between intentional body movements and their outcomes in space. It is therefore plausible to hypothesize that modulations of body schema and peripersonal space similar to those observed here could be found when agents control a virtual or physical object that is not hand-shaped, or not related to the body at all. This is an interesting experimental question that future studies could address.

Body schema provides proprioceptive and somatosensory information about the body morphology during action planning and execution. Theories of motor control and agency suggest that the brain uses internal models and representations to ensure accurate control of movement^44^. According to these views, when a motor command is issued, a “forward model” (or “internal predictive model”) of the moving body estimates the sensory consequence of the action^45^. Sensory information about the body and the environment is then compared with the actual sensory feedback of the action. The result of this comparison is known as prediction error. It is possible to assume that when intentional body movements and their consequences occur synchronously but in different spatial positions, a prediction error is generated. Body schema updating, therefore, reflects the need to achieve control over the body (i.e. an effector) and the environment, minimizing prediction error^46,47^.

It has been theorized and empirically demonstrated, indeed, that forward models predict similar sensory consequences for actions involving a tool, and natural hand movements. In a recent study, it has been found that the predictive attenuation of touch, observed when people touch their hand with the other, is also observed for touches applied with a hand-held tool^48^. Thus, it is possible to assume that the forward model takes into account in its predictions not the location of a body part *per s*e but rather the location of the current effector, i.e. the tip of the tool during tool use in the aforementioned study^48^ or, in our case, a virtual object controlled at distance.

Likewise, peripersonal space is fundamentally a working space used to compute arm and nearby objects positions in order to plan and execute actions. This sector of space is coded by multisensory neurons in the fronto-parietal areas with a tactile receptive field centred on different body parts and a visual and\or an auditory receptive field^9^, partially overlapping with the tactile one. Stimuli from different sensory modalities occurring on or close to the body, are integrated to provide a working space to act on nearby objects (i.e., peripersonal space). In the present study, like body schema, peripersonal space size was updated when hand movements and visual stimuli occurred synchronously but in different spatial positions.

Incidentally, although we highlighted that body schema and peripersonal space show similar plastic effects, we do not claim that they are completely overlapping functional constructs. Indeed, after controlling for the influence of training on peripersonal space, modulations of body schema were still evident. Together with previous evidence^7^, this suggests that plastic changes of body schema and peripersonal space rely, at least in part, on separate mechanisms.

The current findings on peripersonal space are in agreement with previous evidence showing an extension of peripersonal space by using synchronous audio tactile stimuli. Specifically, Serino *et al*.^49^ found that peripersonal space was enlarged after synchronous audio-tactile training, in which hand-tactile stimuli and auditory-far stimuli were simultaneously presented. According to Serino *et al*.^49^ multisensory areas, capturing the synchronicity between the tactile stimulus at the hand and an auditory (or visual) stimulus in the far space, associated the two stimuli, as if they occurred from a functionally equivalent sector of space. However, in Serino *et al*.^49^, participants passively received tactile stimuli on their hand, whereas in the present study participants actively moved their hand, receiving proprioceptive information, and simultaneously perceived a visual stimulus that synchronously responded to their movements in a different position. The current study, therefore, highlights the importance of intentional action to actively create associations between different stimuli occurring in space. The mere occurrence of proprioceptive and visual stimuli *per se*, indeed, is not sufficient to induce changes in body schema and peripersonal space, as demonstrated by the absence of effects in the asynchronous condition. Rather, these changes occur only when the visual stimulus movements followed closely hand movements, i.e., in a strict temporal contiguity (synchronous condition), causing the emergence of a sense of agency over the external event.

It is well known, indeed, that sense of agency depends on processes of temporal associations between an action and its effect. It is possible that in Serino *et al*.^49^ participants developed an implicit sense of agency for the far auditory outcomes, following the audio-tactile training. However, this possibility was not explored in their study.

To sum up, collectively Experiment 1 and Experiment 2 suggest that the experience of controlling external events through one’s own actions is crucial for determining both body schema and peripersonal space extent. This finding opens a new venue into the interpretation of the relationship between body schema, peripersonal space and action. However, future research is needed to understand what are the precise neurocognitive computations involved in controlling an external object, and dynamically updating space and body representations. These findings could have several implications in the field of brain-machine interfaces^50,51^ that enable, trough real time decoding of neural signals, the control of external devices, from robotic arms to virtual avatars.
